# Insights Into the Resistance Mechanisms of Inhibitors to FLT3 F691L Mutation *via* an Integrated Computational Approach

**DOI:** 10.3389/fphar.2019.01050

**Published:** 2019-09-20

**Authors:** Yunfeng Sun, Zhongni Xia, Qinqin Zhao, Bei Zheng, Meiling Zhang, Yin Ying

**Affiliations:** Department of Pharmacy, Tongde Hospital of Zhejiang Province, Hangzhou, China

**Keywords:** AML, FLT3, F691L, molecular modeling studies, resistance mechanism

## Abstract

Research has shown that FMS-like tyrosine kinase 3 (FLT3) may be a vital drug target for acute myeloid leukemia (AML). However, even though the clinically relevant F691L gatekeeper mutation conferred resistance to current FLT3 drug quizartinib, PLX3397 remained unaffected. In this study, the protein–ligand interactions between FLT3 kinase domain (wild-type or F691L) and quizartinib or PLX3397 were compared *via* an integrated computational approach. The classical molecular dynamics (MD) simulations in conjunction with dynamic cross-correlation (DCC) analysis, solvent-accessible surface area (SASA), and free energy calculations indicated that the resistant mutation may induce the conformational change of αC-helix and A-loop of the FLT3 protein. The major variations were controlled by the electrostatic interaction and SASA, which were allosterically regulated by residues Glu-661 and Asp-829. When FLT3-F691L was bound to quizartinib, a large conformational change was observed *via* combination of accelerated MD simulations (aMDs), principal component analysis (PCA), and free energy landscape (FEL) calculations. The umbrella sampling (US) simulations were applied to investigate the dissociation processes of the quizartinib or PLX3397 from FLT3-WT and FLT3-F691L. The calculated results suggested that PLX3397 had similar dissociation processes from both FLT3-WT and FLT3-F691L, but quizartinib dissociated more easily from FLT3-F691L than from FLT3-WT. Thus, reduced residence time was responsible for the FLT3-F691L resistance to inhibitors. These findings indicated that both the conformational changes of αC-helix and A-loop and the drug residence time should be considered in the design of drugs so that rational decisions can be made to overcome resistance to FLT3-F691L.

## Introduction

Acute myeloid leukemia (AML) is the most common type of acute leukemia in adults (De Kouchkovsky and Abdul-Hay, 2016). The clinical symptoms of AML include uncontrolled proliferation, differentiation of immature blast cells in the bone marrow, and impaired production of normal blood cells (De Kouchkovsky and Abdul-Hay, 2016; Behrmann et al., 2018). Patients with AML are routinely profiled for mutations in the tyrosine kinases, phosphatases, and Ras GTPases using genome sequence analysis (Roloff and Griffiths, 2018). Mutations in FMS-like tyrosine kinase 3 (FLT3, also known as CD135) are present in 30–35% of all AML cases during diagnosis. Therefore, FLT3 kinase domain has become a critical therapeutic target in AML (Sutamtewagul and Vigil, 2018; Wu et al., 2018).

**Figure 1:**
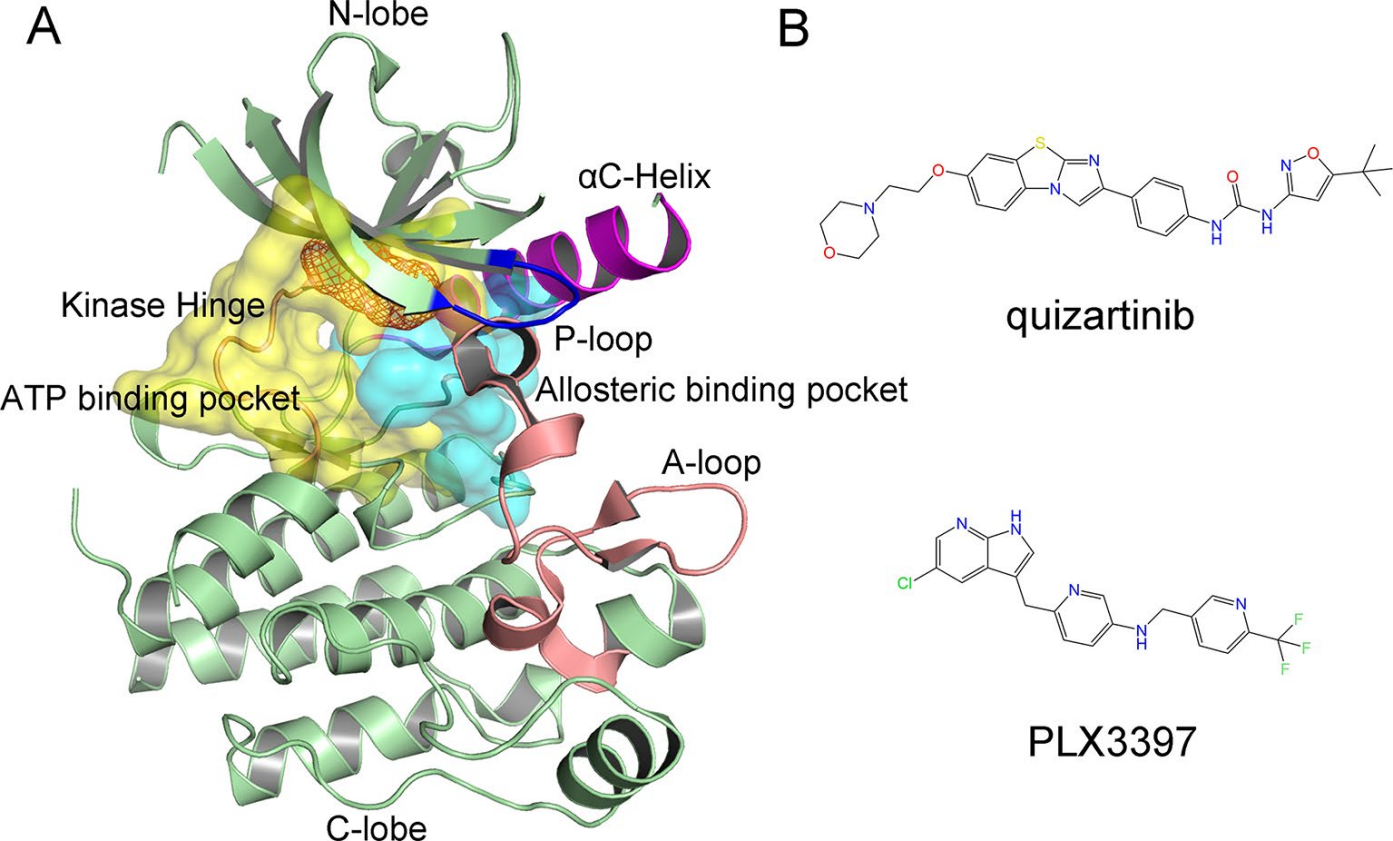
Crystal structure of FLT3 (PDB code: 4RT7) and representative inhibitors. **(A)** Overview of the Mps1 structure, F691L gatekeeper mutation is colored orange dots. The ATP binding pocket is colored yellow surface and allosteric binding pocket is colored cyan surface; **(B)** 2D chemical structures of quizartinib and PLX3397.

The kinase domain of FLT3 consists of a smaller N-terminal lobe and a larger C-terminal lobe, connected by a kinase hinge (**Figure 1A**) (Griffith et al., 2004). The N-terminal lobe features five-stranded anti-parallel β sheets adjacent to the αC-helix. The C-terminal lobe contains seven α helices and three β strands. The critically conserved structural elements for FLT3 catalytic activity lie in between the N-terminal and C-terminal lobes, which include the kinase region, the phosphate-binding loop (i.e., P-loop), the activation loop (i.e., A-loop), the catalytic loop, and the αC-helix. The conserved ATP binding site (yellow surface) and allosteric binding site (cyan surface) are located in the cleft between the two domains occupied by small-molecule inhibitors (**Figure 1A**) (Smith et al., 2015; Zorn et al., 2015).

Several highly potent small-molecule inhibitors of FLT3 have been developed, and some are being evaluated in clinical trials, such as sorafenib, crenolanib, and quizartinib (Garcia and Stone, 2017; Sutamtewagul and Vigil, 2018; Wu et al., 2018). However, FLT3 inhibitors have exhibited antineoplastic activity in patients with relapsed or refractory AML, particularly in patients with FLT3 mutations. To date, resistance to FLT3 inhibitors has been associated with different profiles of FLT3 mutations, including N676K, N676D, F691L, G697R, Y842C/H, and D835H/V/F/I/del/Y (Yamamoto et al., 2001; Heidel et al., 2006; Williams et al., 2013; Sutamtewagul and Vigil, 2018). Among the secondary resistance mutations, the gatekeeper mutation F691L conferred high-level resistance to various promising inhibitors, such as quizartinib (Smith et al., 2015).

Although most of the resistant mutations have been well studied, the resistance mechanisms of FLT3-F691L remain unknown (Swetha et al., 2016; Friedman, 2017; Verma et al., 2018). This study compared quizartinib and PLX3397 to elucidate the drug resistance mechanisms of FLT3-F691L (**Figure 1B**). Quizartinib (formerly known as AC220) is an oral, highly selective and potent, and second-generation FLT3 inhibitor, proven to be more selective and potent than any previous FLT3 inhibitors (Zarrinkar et al., 2009; Smith et al., 2015). PLX3397 is an oral, potent inhibitor with high specificity for colony-stimulating factor-1 receptor (CSF-1R) and has been shown to yield promising therapeutic results in clinical trials (Butowski et al., 2016). Recently, Smith et al. (Smith et al., 2015) reported the first co-crystal structure of FLT3 wild type (WT) with quizartinib, stating that PLX3397 exhibited equivalent activities against FLT3-WT as well as FLT3-F691L. Generally, it was hypothesized that the F691L mutation was located at the gatekeeper and may obstruct key protein–ligand interactions. However, this explanation seems to be relatively ambiguous. Herein, classical molecular dynamics (MD) simulations, accelerated molecular dynamics (aMD) simulations, and umbrella sampling (US) simulations were carried out to shed light on the resistance mechanisms caused by the F691L mutation *via* two promising inhibitors (quizartinib and PLX3397). A combination of classical MD simulations, dynamical cross-correlation (DCC) analysis, and binding free energy calculations were used to help assess the impact of the F691L mutation on the flexibility and the impact of dynamics of FLT3 regions on inhibitors binding. The key protein–ligand interactions related to drug resistance were highlighted by per-residue free energy decomposition. Next, aMD simulations, principal component analysis (PCA), and free energy landscape (FEL) calculations were utilized to achieve enhanced sampling of the conformational space and to elucidate the important collective structural motions. Furthermore, US simulations were performed to analyze the dissociation processes of quizartinib and PLX3397 from the FLT3-WT and FLT3-F691L. These illuminating results deepened our understanding of F691L resistance mechanisms and allowed for better design of novel inhibitors to overcome the F691L gatekeeper mutation of FLT3.

## Methods and Materials

### Preparation of the Initial Complex

The three-dimensional crystal structure of human FLT3-WT + quizartinib (PDB code: 4RT7) was retrieved from the Protein Data Bank (PDB) (Smith et al., 2015). The missing side chains and loop structures of the FLT3 were constructed using the *loops/refine structure* module in UCSF Chimera software (Pettersen et al., 2004). The protonation states of the protein residues were calculated using PDB2PQR Server (Dolinsky et al., 2004). FLT3-F691L was constructed using EasyModeller software (Fiser and Sali, 2003; Kuntal et al., 2010). Then, the modeled structures were refined by UCSF Chimera software, which performed a series of functions, including removing all non-bonded hetero-atoms and water molecules, adding missing hydrogen atoms (Pettersen et al., 2004). The initial coordinates of FLT3-F691L + quizartinib, FLT3-WT + PLX3397, and FLT3-F691L + PLX3397 were obtained from protein–ligand docking studies using the AutoDock software (Morris et al., 2009). A cubic box of 22.5 Å × 22.5 Å × 22.5 Å with a grid spacing of 0.375 Å centered on the binding site was defined. Gasteiger partial charges were assigned to all ligand atoms, and rotatable bonds were identified using AutoDockTools. The affinity maps of FLT3-WT and FLT3-F691L were calculated using AutoGrid software. The docking protocol is as follows: trials of 200 dockings which were clustered according to the RMSD tolerance of 2.0 Å, the maximum number of evaluations set as 25,000,000, and other parameters were set as default. The highest ranked structure for each complex was chosen to conduct further MD simulation protocols. To assess the validity of the docking results, the structures of FLT3-F691L + quizartinib, FLT3-WT + PLX3397, and FLT3-F691L + PLX3397 were simulated and compared to the corresponding crystal structure.

### Classical MD Simulation and Calculation

Classical MD simulations were performed to examine the quizartinib and PLX3397 association with FLT3-WT and FLT-F691L using Assisted Model Building with Energy Refinement (Amber) 14 software. The partial atomic charges for quizartinib and PLX3397 were calculated by using the restrained electrostatic potential (RESP) method based on HF/6-13G* basis set. Then, the proteins and ligands were described by the Amber ff14SB force field and General Amber Force Field (GAFF) by LEaP modules in Amber 14 software, respectively (Wang et al., 2004; Maier et al., 2015). Thereafter, protein–ligand systems were solvated in a cubic box of TIP3P water molecules with a 20 Å distance between the complex surface and the box boundary. To ensure the overall charge neutrality, an appropriate number of counter ions were added.

Prior to classical MD simulation, a sophisticated protocol was performed, including three-step minimizations, heating, and equilibration. During the three-step minimizations, solvent atoms were first held fixed while solute molecules were relaxed. Next, solute atoms were held fixed while solvent molecules were relaxed. The last step was to allow all atoms to move freely without any restraint. During each step, 2,500 steps of steepest descent algorithm and 2,500 steps of conjugated gradient algorithm were applied. After that, the system was heated up from 0 to 310 K in 100 ps using a Langevin thermostat with harmonic restraints of 3 kcal mol^−1^ Å^−2^, followed by the density procedure which was applied at 310 K for 200 ps and equilibration for 1 ns in the isothermal isobaric (NPT) ensemble. Finally, the system was submitted to 500-ns classical MD simulation in the NPT ensemble. During simulations, nonbonded terms were calculated with a 8.0 Å cutoff, and long-range electrostatics were treated by particle-mesh Ewald (PME) algorithm (Essmann et al., 1995). The temperature was maintained at 310 K and pressure at 1 bar using Langevin dynamics (collision frequency [γ] 2.0) and Berendsen barostat, respectively (Izaguirre et al., 2001). Covalent bonds connecting hydrogen atoms were constrained with the SHAKE algorithm (Krautler et al., 2001). A time step of 2 fs was performed and coordinates were recorded every 10 ps for further analysis.

### DCC Analysis

To gain better insights into the dynamics of the structure of the simulated systems, DCC map (DCCM) was generated to analyze the cross-correlation shifts of the backbone atoms (C_α_). The cross-correlation matrix (*C* *_ij_*) between residues *i* and *j* were calculated based on 300–500-ns classical MD simulation trajectories with a total of 1,000 snapshots by using the Bio3D package of R (Skjaerven et al., 2014). The *C* *_ij_* is determined by the following equation:

(1) $$C_{\mathrm{ij}}=\frac{\langle ∆r_{i\cdot }∆r_{j}\rangle }{\sqrt{\langle ∆r_{i}^{2}∆r_{j}^{2}\rangle }}$$

where Δ*r* *_i_* or Δ*r* *_j_* is the displacement from the mean position of the *i* ^th^ or *j* ^th^ atom, and angle bracket denotes an average over the sampled period.

### Classical MD Simulation Based Binding Free Energy Calculations

One of the widely accepted methods to calculate the binding free energy (Δ*G*_binding_) of a non-covalently bound small molecule to protein is molecular mechanics (MM)/Poisson–Boltzmann surface area (PBSA) (Sun et al., 2014; Genheden and Ryde, 2015). The Δ*G*_binding_ is given by:

(2) $$G_{binding}=G_{\text{Receptor}+\text{Ligand}}-(G_{\text{Receptor}}+G_{\text{Ligand}})$$

The free energy term is calculated as an average over the considered structures (in this study, an ensemble of time-equidistant snapshots):

(3) $$\langle G\rangle =\langle E_{\mathrm{MM}}\rangle +\langle G_{\mathrm{sol}}\rangle -T\langle S_{\mathrm{MM}}\rangle$$

The energetic term *E*_MM_ is defined as:

(4) $$E_{\mathrm{MM}}=E_{\mathrm{int}}+E_{\mathrm{vdW}}+E_{\mathrm{elec}}$$

(5) $$G_{\mathrm{sol}}=G_{\mathrm{polar}}+G_{\mathrm{nonpolar}}$$

*E*_MM_ can be split into three terms (Eq. 4): intermolecular interaction energy (*E*_int_), van der Waals energy (*E*_vdW_), and electrostatic energy ∆*E*_elec_). The solvation term *G*_solv_ can be split into polar (*G*_polar_) and nonpolar contributions (*G*_nonpolar_). The *G*_polar_ represents the energy required to transfer the solute from a continuum medium with a low dielectric constant (ε = 1) to a continuum medium with the dielectric constant of water (ε = 80). *G*_polar_ was estimated using the nonlinearized Poisson Boltzmann equation. The *G*_nonpolar_ is calculated using the solvent accessible surface area (SASA) model. Conformational entropy was not calculated because of the high computational demand and low prediction accuracy (Hou et al., 2011). In this study, the ∆*G*_binding_ is calculated by using the trajectories from classical MD simulations between 300 and 500 ns with 1,000 snapshots.

### Accelerated MD (aMD) Simulations

The equilibrated snapshots provided by classical MD simulations were used as the initial conformations for the aMD simulations. Dual-boost aMD simulations were performed in our study, which means that both the total potential energy and the dihedral energy of the system were added as the following Equations:

(6) $$E_{\mathrm{total}}=V_{\mathrm{total}\_\mathrm{avg}}+(n\times a{\text{​}}_{\mathrm{total}}),\;a_{\mathrm{total}}=0.2\times N_{\mathrm{atoms}}$$

(7) $$E_{\mathrm{dihed}}=V_{\mathrm{dihed}\_\mathrm{avg}}+3.5\times N_{\mathrm{residues}}$$

where *V*_total_avg_ and *V*_dihed_avg_ are time averages of the total potential energy and dihedral energy obtained from classical MD simulation. The parameters *N*_atoms_ and *N*_residues_ are the number of atoms in the system and the number of protein residues. In eq.6, n (1,2,3…) is an integer that determines the magnitude of the threshold as a multiple of the acceleration factor. In this study, a classical MD simulation was carried out for 20 ns to obtain the average total potential energy and the average dihedral potential energy before the aMD simulations. Then, 600-ns aMD simulations were performed. During the simulations, PME algorithm was applied to evaluate the long-range electrostatic interactions, and the nonbonded interactions were truncated at 10.0 Å. The hydrogen atoms involved in covalent bonds were regulated by the SHAKE algorithm (Krautler et al., 2001). The temperature was maintained at 310 K using a Langevin thermostat with a coupling constant of 2.0 ps^−1^. The coordinates were saved every 4 ps, and numerical analysis was performed with CPPTRAJ module in Amber 14 (Roe and Cheatham, 2013).

### Principal Component Analysis (PCA) and Free Energy Landscape Calculations for the aMD Simulations

To identify the differences of collective motion between the simulated systems, PCA were performed using with CPPTRAJ module in Amber 14. After solvent and ions were first stripped off, PCA was performed on the whole trajectories from aMD simulation. Thereafter, the potential boost in conjunction with the principal component 1 (PC1) and principal component 2 (PC2) calculated from PCA were used to recover the FEL by the cumulant expansion to the second order method (Roe and Cheatham, 2013; Miao et al., 2014).

### Umbrella Sampling (US) Simulations

The equilibrated snapshots provided by the trajectories from classical MD simulations were used as the initial structures for the US simulations. The largest pocket direction was set as the initial reaction coordinates (RCs) for each system, which was identified by using the CAVER Analyst 1.0 software (Kozlikova et al., 2014). Then, to quantificationally define the unbinding pathway, the distance between one atom in receptor (C_α_ in Thr-666 in FLT3 WT/F691L) and another nitrogen atom in ligand was selected as the RCs for quizartinib (**Figure S1A**). For PLX3397, the distance between one atom in receptor (C_α_ in Met-664 in FLT3 WT/F691L) and another carbon atom in ligand was chosen as the RCs (**Figure S1B**). In the present study, the RCs of these systems were extended 25 Å from the initial distance unbinding from ATP pathway. In each unbinding pathway, the RCs were separated into 51 windows by a step of 0.5 Å, and initial conformation for each window was formed last snapshot of the previous window. As an example, the initial conformation of window 10 was from the last snapshot of the window 9. For each window, 10-ns US simulations were performed to each window in order to ensure the convergence of each system. Besides, an elastic constant of 10 kcal/mol/Å^2^ was employed in all of the US simulation windows to pull each ligand away from the binding cavity at a constant force and speed with a total of 2.04-μs US simulations being performed. The weighted histogram analysis method (WHAM) was utilized to calculate the potential of mean force (PMF) along the RC (Kumar et al., 1992). For WHAM calculation, the RC was split into 400 bins, and tolerance for iteration was set at 0.0001.

## Results and Discussion

### Evaluation of the Stability of the Simulated Systems

Structural alignments of the docking models of FLT3-F691L + quizartinib, FLT3-WT + PLX3397, and FLT3-F691L + PLX3397 with their corresponding crystal structures were performed to verify the docking results. As shown in **Figure S2A**, alignment of the model structure of FLT3-F691L + quizartinib model structure to the FLT3-WT+quizartinib crystal structure (PDB code: 4RT7) exhibited high similarity, with a root-mean-square deviation (RMSD) of 4.82 Å for heavy atoms. The alignment of FLT3-WT + PLX3397 and FLT3-F691L + PLX3397 models to the crystal structure of CSF-1R-WT + PLX3397 (PDB code: 4R7H) showed many similarities with minor differences (**Figure S2B**). These predicted conformations were used for the subsequent MD simulations.

In order to investigate the dynamic features of FLT3-WT + quizartinib, FLT3-F691L + quizartinib, FLT3-WT + PLX3397, and FLT3-F691L + PLX339, classical MD simulations were performed for 500 ns. Prior to analysis of the MD simulation data, the water molecule in the crystal structure of FLT3-WT + quizartinib was checked due to it not being considered in the molecular docking study for construction of FLT3-F691L + quizartinib. As shown in **Figure S3**, the water molecule was located between the hinge region and quizartinib in both FLT3 WT and F691L, which was near the water molecule in the crystal structure. This finding indicated that this water molecule was not the determining factor of drug resistance for our subsequent analysis.

**Figure 2:**
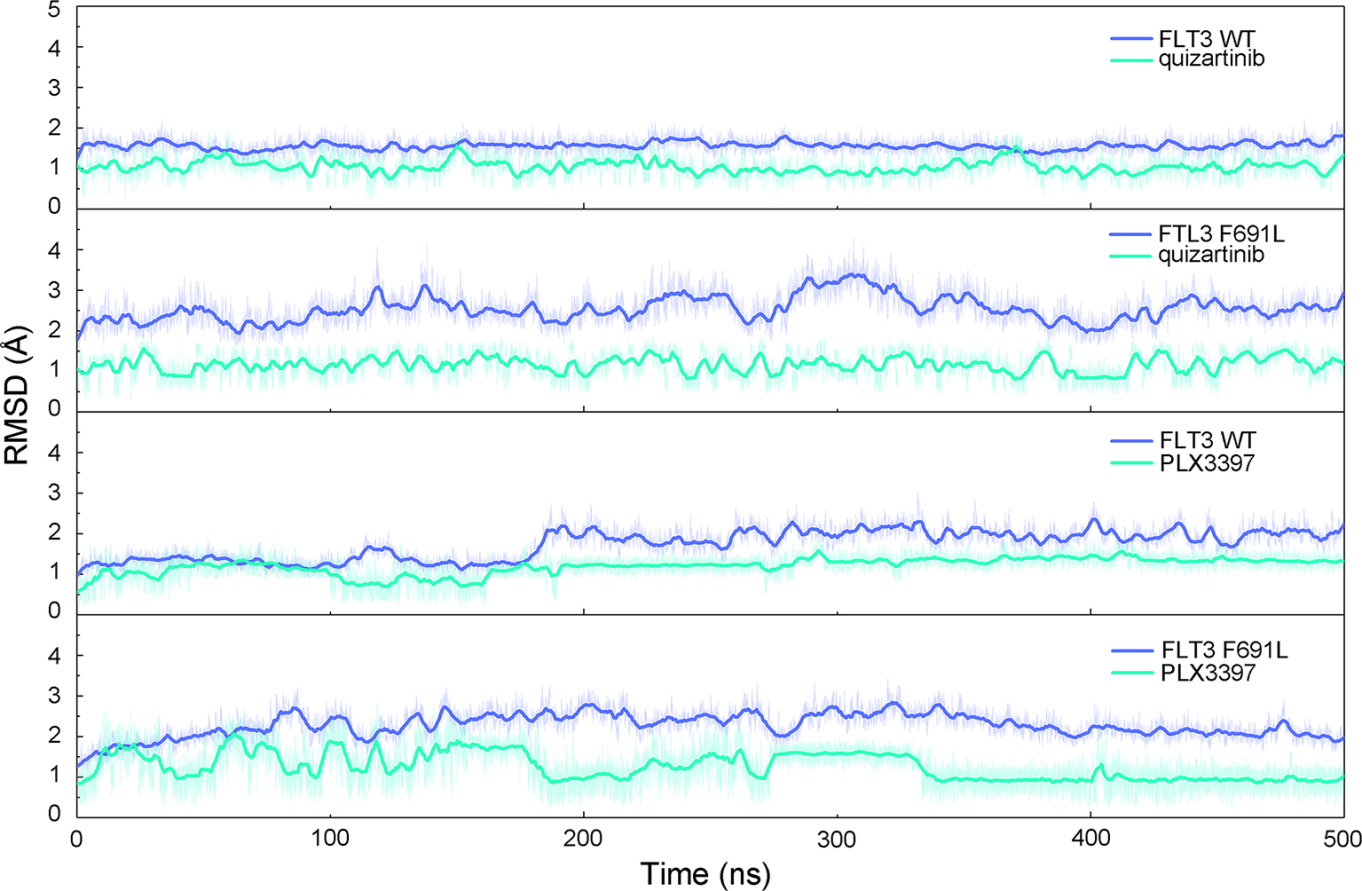
RMSD values of the backbones of proteins and heavy atoms of ligands in the process of classical MD simulations.

The stability of the systems was a prerequisite for all further analyses. Therefore, RMSDs of protein backbones and heavy atoms of ligands were applied to monitor the dynamic stability of the studied systems during classical MD simulations. The smaller the fluctuations of RMSDs, the better the stability of the protein–ligand structure. As plotted in **Figure 2**, the RMSDs of protein backbones and heavy atoms of the ligands in all systems quickly reached a steady state after approximately 10–300 ns of classical MD simulation. The RMSD curves of FLT3-WT protein backbone for quizartinib oscillated with minute fluctuations (less than 1 Å), indicating that quizartinib constrained the structural flexibility of FLT3-WT. However, the RMSD curves of both FLT3-F691L protein and quizartinib ligand fluctuated more than the FLT3-WT, as shown in **Figure 2**. This indicated the unstable nature of FLT3-F691L when it was bound to quizartinib. The RMSD curves for PLX3397 in both FLT3-WT and FLT3-F691L oscillated with minute fluctuations (less than 1 Å) during the last 200-ns simulation demonstrated that PLX3397 constrained the structural flexibility of both FLT3-WT and FLT3-F691L. Thus, the structural and energetic properties of complexes formed between quizartinib and PLX3397 with FLT3-WT and FLT3-F691L were analyzed during the last 200-ns classical MD simulation trajectories.

### Interregional Correlated Motions of the Simulated Systems

**Figure 3:**
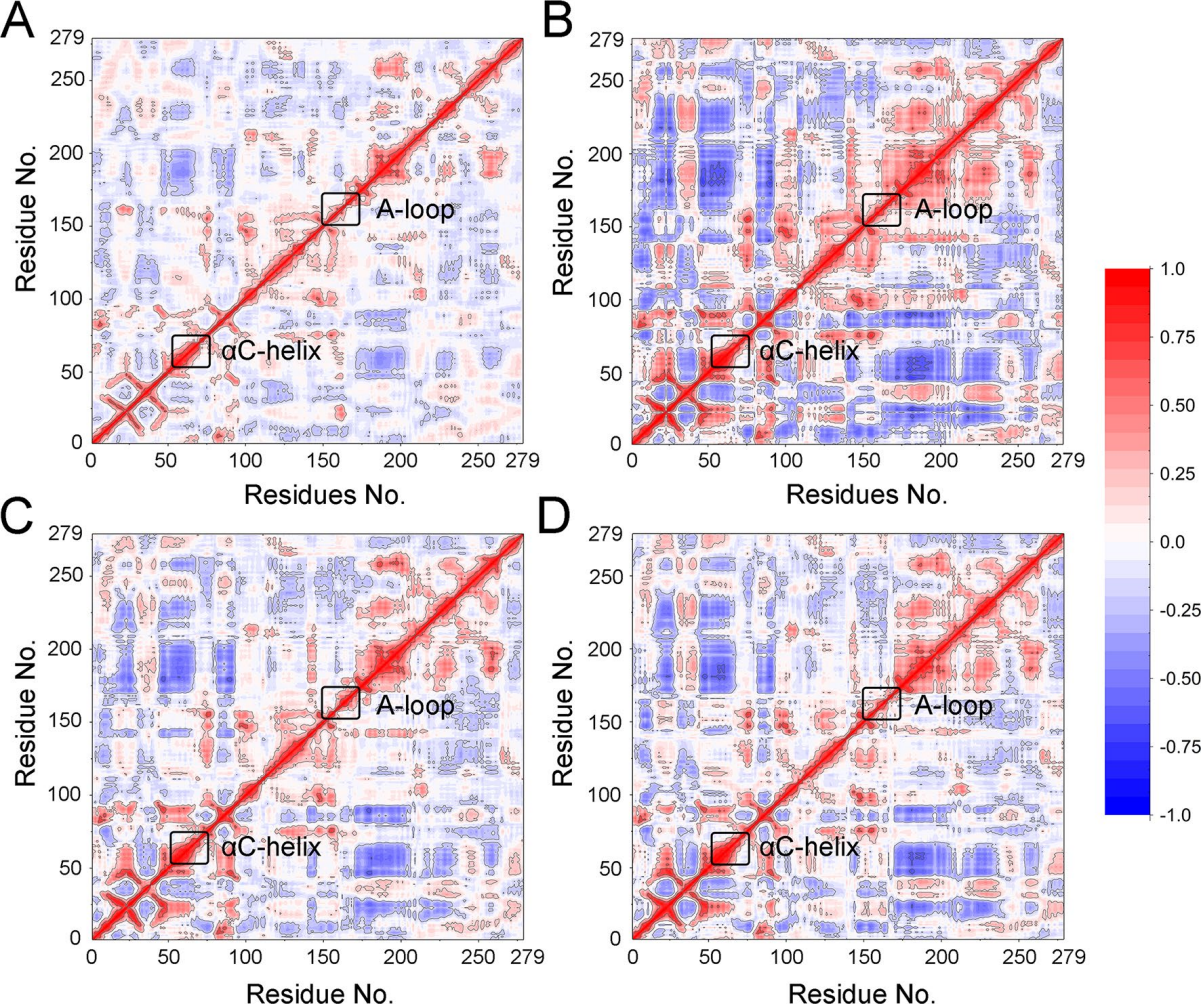
DCCM from classical MD simulations. **(A)** FLT3-WT + quizartinib; **(B)** FLT3-F691L + quizartinib; **(C)** FLT3-WT + PLX3397; **(D)** FLT3-F691L + PLX3397.

**Figure 4:**
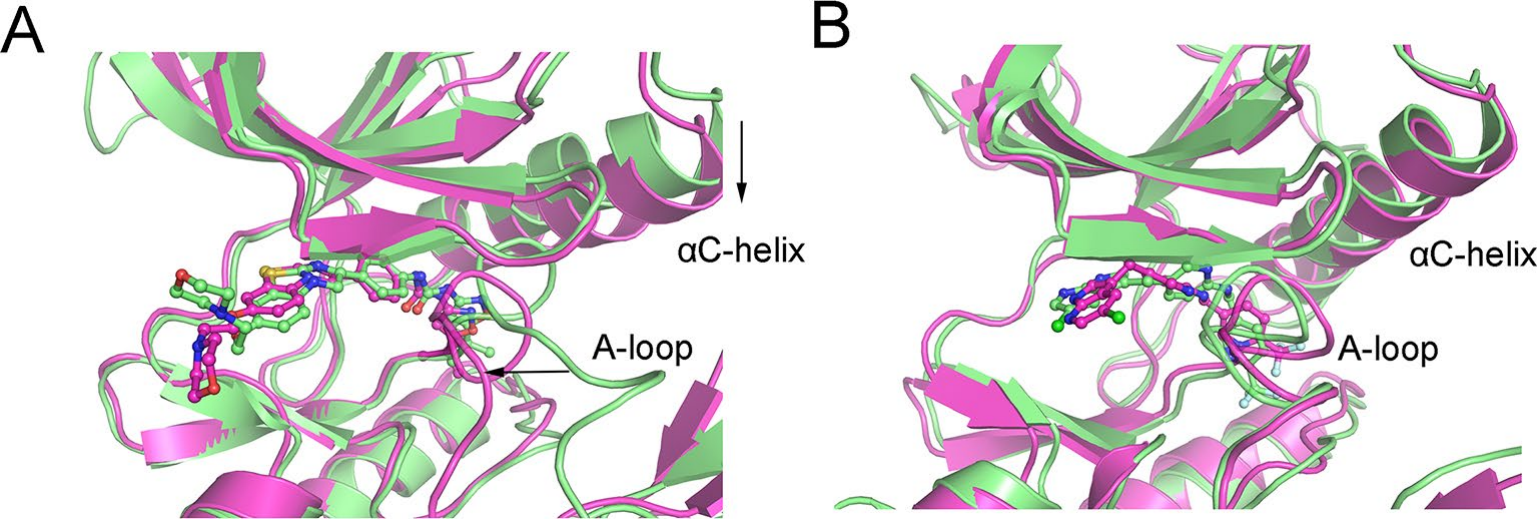
Alignment of the most populated structures between **(A)** FLT3-WT + quizartinib and FLT3-F691L + quizartinib; **(B)** FLT3-WT + PLX3397 and FLT3-F691L + PLX3397.

DCC map (DCCM) is one of the most popular methods used to analyze the conformational dynamics of proteins in two dimensions (Ichiye and Karplus, 1991; Wang et al., 2018). As shown in **Figure 3**, the correlation coefficients range from −1 to +1, corresponding to three color representations: red color represented positive correlation values ranging from 0.25 to 1, blue color represented anti-correlation values ranging from −0.25 to −1, and light red or blue color represented weak or no-correlation for values ranging from −0.25 to + 0.25. The extent of correlation or anti-correlation is indicated by the respective color intensities. As illustrated in **Figures 3A, B**, both the red and blue regions in the FLT3-F691L + quizartinib were larger and more intense than that of FLT3-WT + quizartinib, implying that the correlation or anti-correlation motions were enhanced in the FLT3-F691L. On the contrary, color regions for FLT3-WT and FLT3-F691L bound with PLX3397 were quite similar (**Figures 3C, D**). In addition, important areas marked with black boxes showed clear differences between the systems. In the FLT3-F691L + quizartinib, the αC-helix, and A-loop exhibited amplified motions, whereas the dynamics of the other three systems were slightly correlated. Alignment of the most populated structures showed that the αC-helix of FLT3-F691L + quizartinib changed into downward-moving and A-loop region changed into an inward-moving conformation compared with FLT3-WT + quizartinib (**Figure 4A**) but were similar between FLT3-WT/PLX3397 and FLT3-F691L/PLX3397 (**Figure 4B**). These observations indicated that the relative motions of different sub-domains, especially the essential αC-helix and A-loop sub-domains, may affect the stability and the accessibility of protein to solvent.

**Figure 5:**
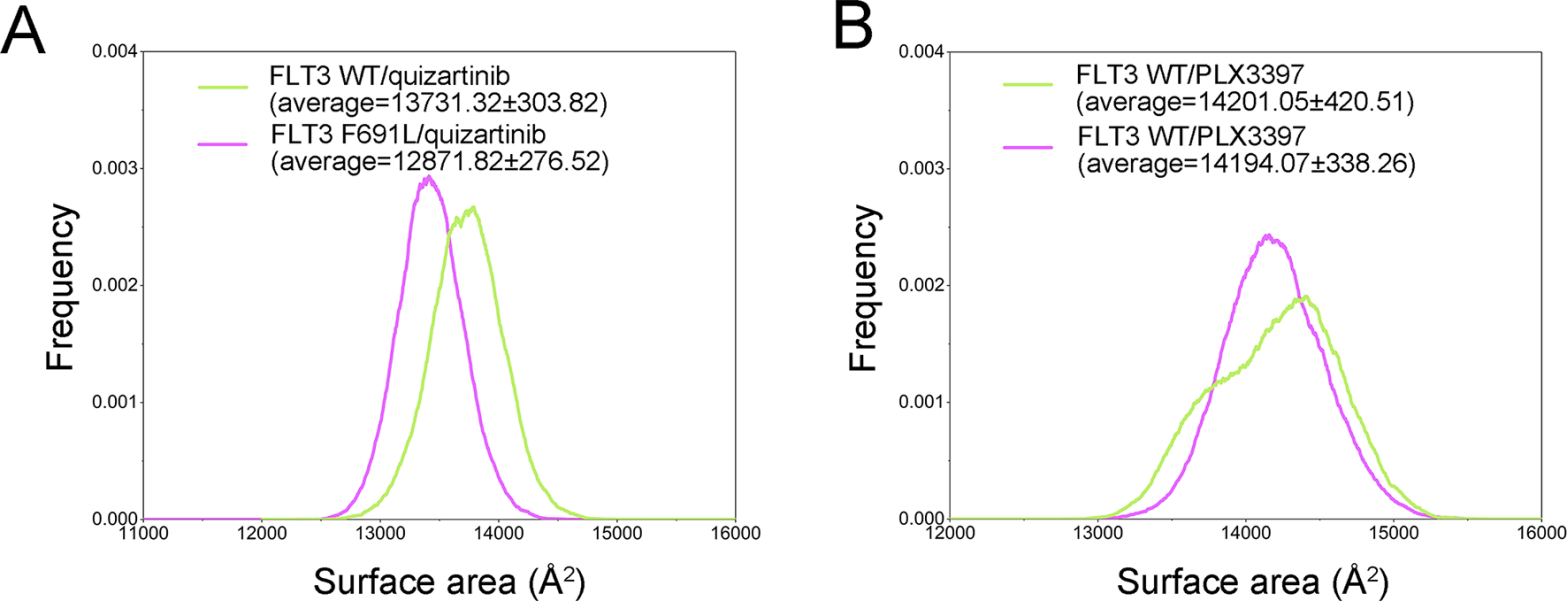
The distribution of solvent-accessible surface area (SASA). **(A)** FLT3-WT + quizartinib and FLT3-F691L + quizartinib; **(B)** FLT3-WT + PLX3397 and FLT3-F691L + PLX3397.

Theoretically, hydrophobic interactions between non-polar amino acids confer stability to proteins in solution by shielding the non-polar amino acids in hydrophobic cores away from the aqueous environment (Bellissent-Funel et al., 2016). In this study, solvent SASA was calculated to highlight the accessibility of proteins to solvent. As shown in **Figure 5A**, the FLT3-WT + quizartinib (13,741.32 ± 303.82 Å^2^) showed a larger average SASA than the FLT3-F691L + quizartinib (12,871.82 ± 276.52 Å^2^), indicating that FLT3-F691L bound with quizartinib decreased the SASA resulting in protein instability. As a comparison, the FLT3-WT and FLT3-F691L bound with PLX3397 (14,201.05 ± 420.51 Å^2^ *versus* 14,194.07 ± 338.26 Å^2^, respectively) showed a very similar distribution (**Figure 5B**). These observations are in agreement with the RMSDs and DCCM, whereby the stability of the FLT3 with quizartinib complex was weakened in the presence of F691L mutation. However, these conformational changes were not observed in the complexes of FLT3-WT or FLT3-F691L bound to PLX3397. Overall, these findings indicated that F691L mutation-induced conformational change might be the main driving force for energy redistributions.

### Binding Free Energies Predicted by MM/PBSA

MM/PBSA binding free energy calculations were employed to assess the energy aspects of quizartinib and PLX3397 when they were associated with FLT3-WT or FLT3-F691L. This method has been widely employed to assess protein–ligand interactions and have provided reliable information regarding the effects of protein mutations on the binding affinities toward diverse ligands (Sun et al., 2014; Genheden and Ryde, 2015). In this study, the binding free energies (∆*G*_binding_) from the decomposition for all systems were analyzed to determine the energy contributions in the receptor-ligand complexes. As reported in **Table 1**, the predicted ∆*G*_binding_ for FLT3-WT + quizartinib, FLT3-F691L + quizartinib, FLT3-WT + PLX3397, and FLT3-F691L + PLX3397 were −18.00 ± 2.45, −6.35 ± 2.89, −9.22 ± 1.63, and −9.16 ± 1.72 kcal/mol, respectively. As expected, the ∆*G*_binding_ of FLT3-WT + quizartinib was lower than FLT3-F691L + quizartinib but was similar when bound to PLX3397. The ∆*G*_binding_ show correlation with the above findings. Herein, only the electrostatic interactions (∆*E*_elec_) and nonpolar contribution to solvation term (∆*G*_SA_) were discussed. The decreased ∆*E*_elec_ and increased ∆*G*_SA_ both led to the decrease of the binding free energy between FLT3-F691L and quizartinib compared to FLT3-WT and quizartinib. The ∆*E*_elec_ and ∆*G*_SA_ between PLX3397 and FLT3-WT or FLT3-F691L showed no difference.

**Table 1:** Binding free energies of quizartinib and PLX3397 in FLT3-WT and FLT3-F691L (kcal/mol).

	WT/quizartinib	F691L/quizartinib	WT/PLX3397	F691L/PLX3397
∆*E*_vdW_	−70.64 ± 3.38	−71.09 ± 3.29	−50.28 ± 3.50	−51.56 ± 3.22
∆*E*_elec_	−35.72 ± 2.31	−30.26 ± 1.62	−97.26 ± 1.40	−96.49 ± 1.21
∆*G*_GB_	54.15 ± 3.78	54.43 ± 2.21	111.65 ± 2.40	112.05 ± 3.57
∆*G*_SA_	33.76 ± 1.40	38.45 ± 1.26	26.67 ± 1.18	26.85 ± 0.73
∆*G*_binding_	−18.45 ± 2.45	−8.35 ± 2.89	−9.22 ± 1.63	−9.16 ± 1.72
Δ*W*_PMF_	−23.43 ± 0.47	−12.99 ± 0.55	−15.80 ± 0.47	−15.36 ± 0.39

*∆E* *_vdW_* *, van der Waals energy; ∆E* *_elec_* *, electrostatic energy; ∆G* *_GB_* *, electrostatic contribution to solvation; ∆G* *_SA_* *, non-polar contribution to solvation; ∆G* *_binding,_* *binding free energy; ΔW* *_PMF,_* *binding affinity based on 20–25 Å along the RC.*

**Figure 6:**
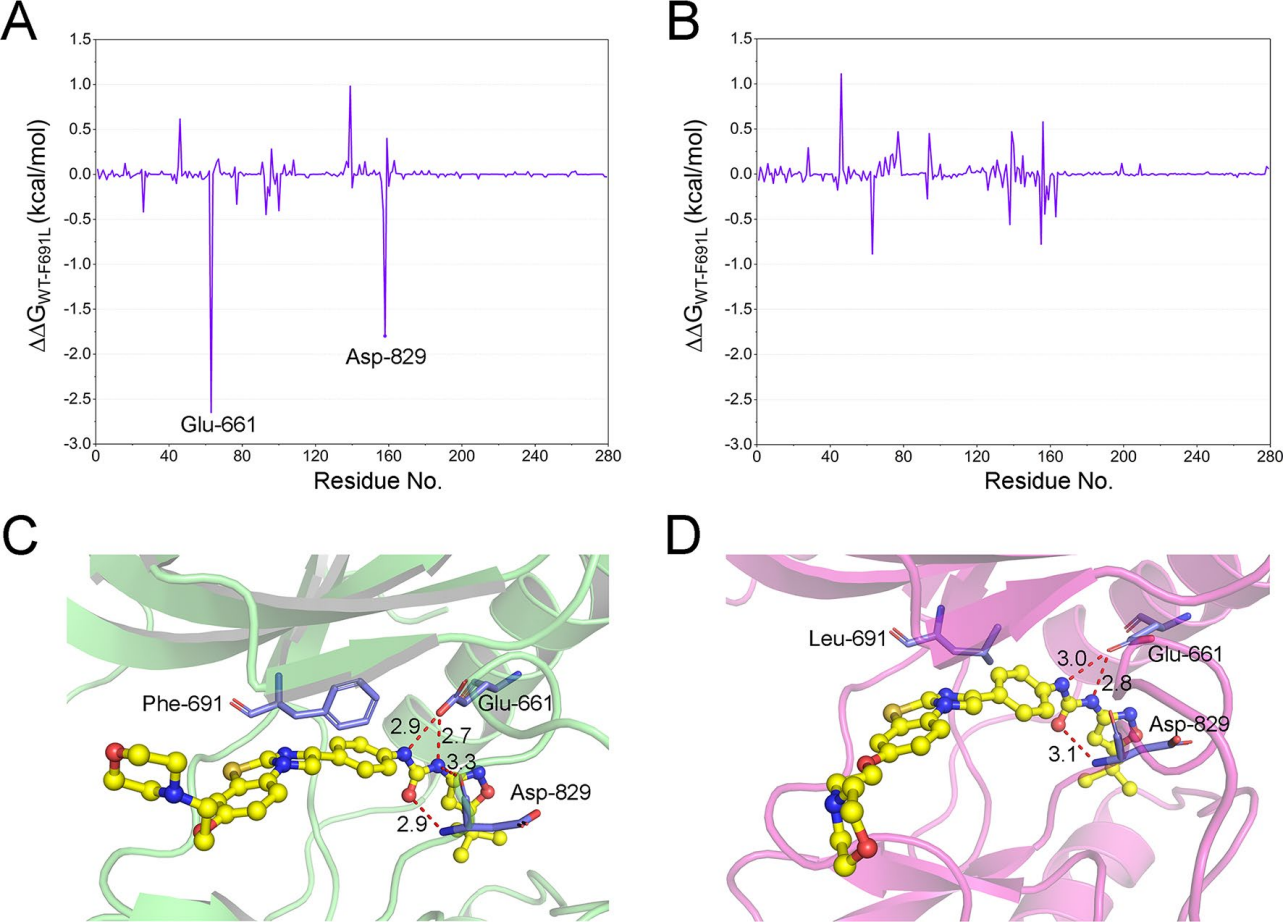
The energetic differences of the residue contributions to the binding free energies between the WT system and F691L system (∆∆*G*=∆*G*_WT_ − ∆*G*_F691L_). **(A)** FLT3-WT + quizartinib and FLT3-F691L + quizartinib; **(B)** FLT3-WT + PLX3397 and FLT3-F691L + PLX3397; **(C)** representative structure of FLT3-WT + quizartinib; **(D)** representative structure of FLT3-F691L + quizartinib. Hydrogen bonds are colored red, and the key residues are colored purple.

Further in-depth analysis of the resistance mechanism was carried out to assess the average energy contributions to the interactions of the complexes *via* decomposition of residue-ligand pairs based on MM/PBSA method. Then, energy differences of per-residue between WT and mutant systems (∆∆G = ∆G_WT_ − ∆G_F691L_) were plotted to indicate the key residues (**Figures 6A, B**). The negative values suggested that the residues of the FLT3-WT formed stronger interactions with ligand than the FLT3-F691L, while positive values indicated that the residues of FLT3-F691L formed stronger interactions with ligand than with the FLT3-WT. As illustrated in **Figure 6A**, the residues of Glu-661 and Asp-829 demonstrated significantly stronger interactions with quizartinib in the FLT3-WT than in FLT3-F691L. Meanwhile, the differences between FLT3-WT + PLX3397 and FLT3-F691L + PLX3397 were quite small. Notably, the Glu-661 and Asp-829 residues are located in the two conserved structural elements of αC-helix and A-loop required for kinase catalytic activity, between the N-terminal and the C-terminal lobes (**Figures 6C, D**). The impact of F691L mutant on nearby residues appeared to be subtle. Structural analysis of FLT3-WT + quizartinib revealed that the carbonyl group of urea formed hydrogen bonds with the backbone nitrogen of Asp-829 at a distance of 2.9 Å. In addition, the nitrogen in the urea moiety formed three hydrogen bonds with two backbone carbonyl groups of Asp-829 and side chain of Glu-661 with distances of 3.0, 2.8, and 3.3 Å, respectively (**Figure 6C**). Binding of quizartinib with FLT3-F691L exhibited different phenomenon (**Figure 6D**). One of the hydrogen bonds between nitrogen in the urea moiety and backbone carbonyl of Asp-829 disappeared. In addition, the length of remaining hydrogen bonds extended from 0.1 to 0.2 Å, indicating that the hydrogen-bond networks for quizartinib bound to FLT3-F691L were less stable than the networks bound to FLT3-WT, which is consistent with the results of the energy analysis.

### aMD Simulations

**Figure 7:**
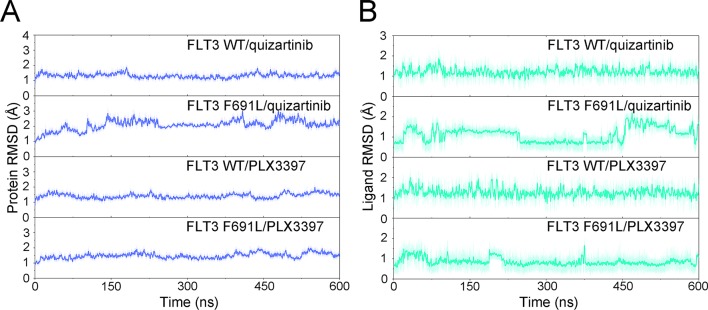
RMSD values of quizartinib and PLX3397 in FLT3-WT and FLT3-F691L from aMD simulations. **(A)** RMSD values of the backbones of proteins; **(B)** RMSD values heavy atoms of ligands.

The aMD simulation provided the possibility for sampling conformational ensembles in greater detail and detection of possible energy barriers that remained hidden in the classical MD simulations. Previous studies suggested that aMD simulation can boost the conformational sampling by up to 2,000 times compared with classical MD simulation (Hamelberg et al., 2004; Bal and Neyts, 2015; Miao and McCammon, 2018). Therefore, aMD simulation was applied to further explore conformational ensembles. Following aMD simulations, the RMSDs of the protein backbone and the heavy atoms of ligand were calculated. As plotted in **Figure 7A**, the RMSDs of protein backbones in all the systems achieved equilibrium after 150 ns of aMD simulations. **Figure 7B** showed that the RMSDs of the heavy atoms of quizartinib and PLX3397 in each system maintained dynamic constant during the 600 ns of aMD simulation. In addition, the fluctuations of quizartinib bound to FLT3-WT were lower than when bound to FLT3-F691L. But when it was bound to PLX3397, the results showed similar findings to the classical MD simulation. The above findings suggested that the F691L mutation allowed for larger conformational changes and more variability among protein subunits.

**Figure 8:**
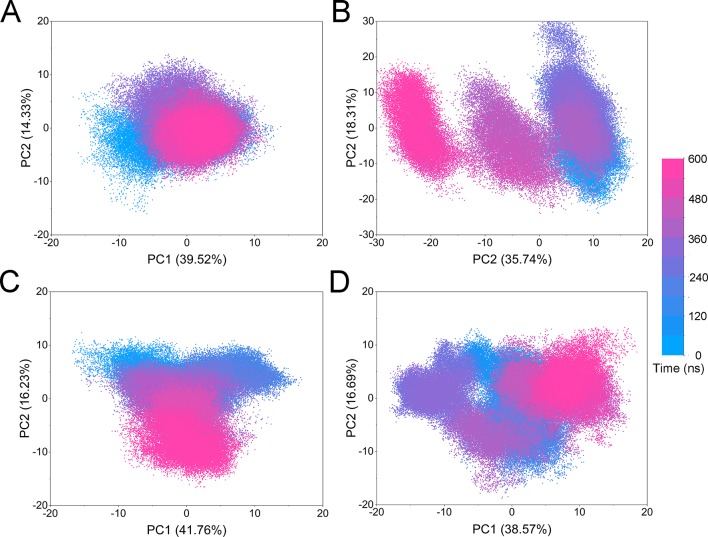
Principal component distributions from aMD simulations. **(A)** FLT3-WT + quizartinib; **(B)** FLT3-F691L + quizartinib; **(C)** FLT3-WT + PLX3397; **(D)** FLT3-F691L + PLX3397.

PCA was used to further characterize the conformational transitions over time (**Figure 8**). PCA, one of the most commonly used statistical methods to explore the differences in biological systems, is used to distinguish the collective motions from the local dynamics and extract the collective motions (Czelleng et al., 1987; Tripathi et al., 2016). Theoretically, the first two eigenvectors (PC1 and PC2) captured most of the variance in the original distribution of the protein conformational ensembles. As shown in **Figure 8**, the PC1 and PC2 explains more than 50% of the motions, suggesting PC1 and PC2 could represent the protein conformational ensembles. The conformation transitions of the simulated systems were analyzed by projecting the trajectories of the principal components (PC1 and PC2) onto the two-dimensional space image. When principal components are plotted against each other, highly similar structures are clustered, hence, each cluster represents a different protein conformational state. As plotted in **Figure 8**, the conformations of all the simulated systems were dynamic and fluctuant during 0–600-ns aMD simulations, and eventually, stabilized in one dominating state. The conformational clusters demonstrated that the conformational distributions of FLT3-WT + quizartinib were remarkably different from those of FLT3-F691L + quizartinib (**Figures 8A, B**), while those for FLT3-WT + PLX3397 and FLT3-F691L + PLX3397 were also different (**Figures 8C, D**). Notably, the range of the conformational distributions for FLT3-F691L + quizartinib were much wider than those for FLT3-WT + quizartinib, while those for FLT3-WT + PLX3397 and FLT3-F691L + PLX3397 were similar to a certain degree. The PCA results revealed that quizartinib with FLT3-WT had a different conformational flexibility compared to FLT3-F691L, as indicated by the RMSD results from both classical and accelerated MD simulations.

**Figure 9:**
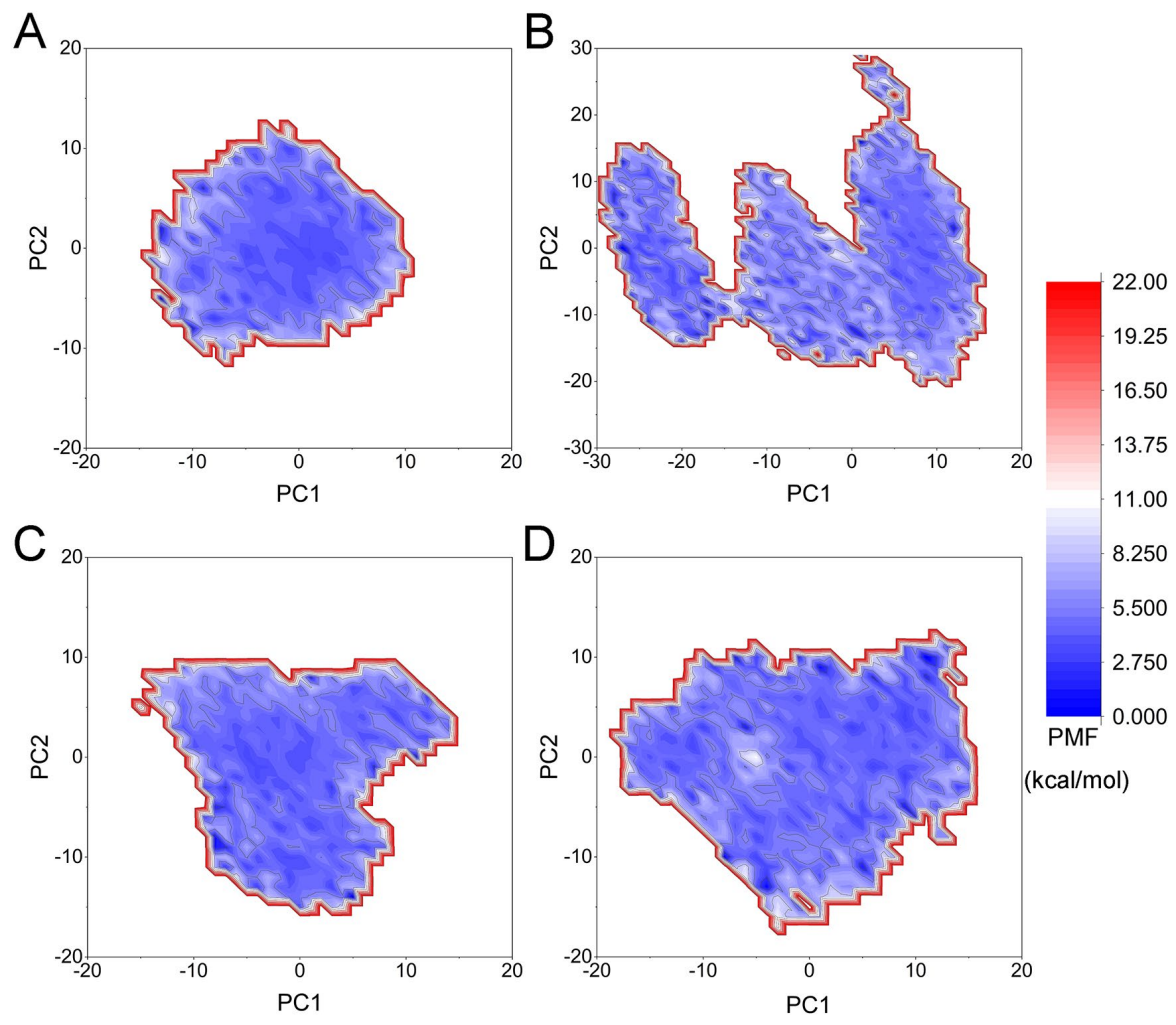
FEL from the aMD simulations. **(A)** FLT3-WT + quizartinib; **(B)** FLT3-F691L + quizartinib; **(C)** FLT3-WT + PLX3397; **(D)** FLT3-F691L + PLX3397.

The FEL was used to assess the relationship between the conformational changes and energy changes (**Figure 9**). More energy wells (dark blue regions) indicated that the protein underwent larger conformational changes during simulation. As shown in **Figure 9A**, the quizartinib bound to FLT3-WT was confined to a single deep energy well throughout the simulation. However, more than two deep energy wells were observed for the FLT3-F691L system (**Figure 9B**), highlighting the unstable nature of FLT3-F691L bound to quizartinib during aMD simulations. Both FLT3-WT and FLT-F691L bound to PLX3397 were quite similar. These observations supported the formerly presented results of energy differences of per-residue between the WT and mutant systems, where Glu-661 and Asp-829 residues may allosterically regulate the conformational ensemble to rendering resistance to quizartinib.

### Unbinding Pathways of Quizartinib and PLX3397 Dissociating From FLT3-WT and FLT3-F691L

**Figure 10:**
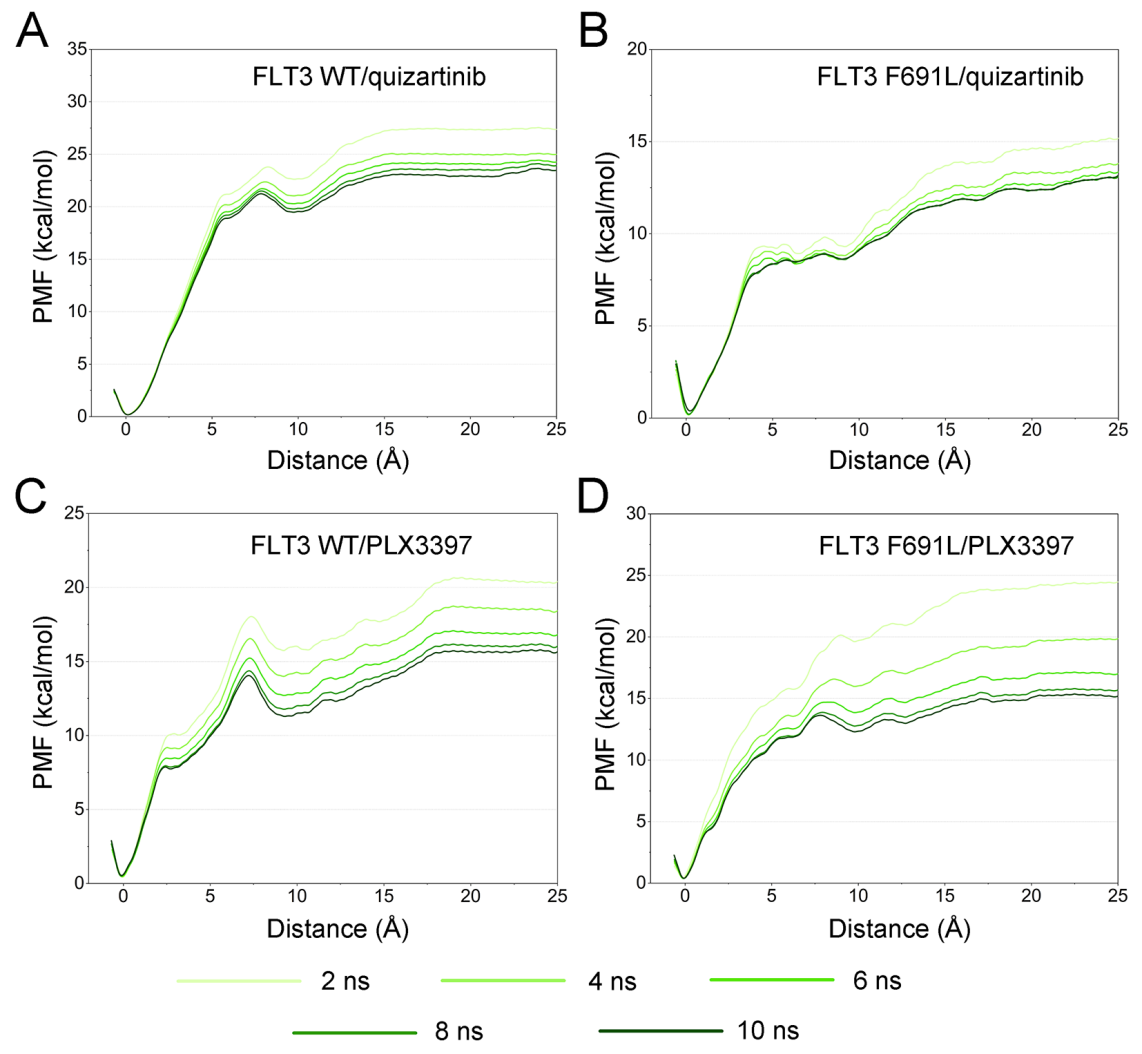
Convergence of the PMFs calculated for four systems from US simulations. **(A)** FLT3-WT + quizartinib; **(B)** FLT3-F691L + quizartinib; **(C)** FLT3-WT + PLX3397; **(D)** FLT3-F691L + PLX3397.”

To ensure the sampling convergence of the US simulations, 10-ns US simulation was carried out for each window, and the convergence of PMF was checked every 2 ns. As shown in **Figure 10**, five curves were plotted for each system, and all of the converged PMFs exhibited no obvious rising at ∼20 Å along RCs. These findings indicated that the PMFs achieved satisfactory coincidence after 8-ns US simulations for each window (difference of PMFs, 0.5 kcal/mol), and the length of 25 Å for RCs was suitable for ligands out of the binding pocket. The binding affinities (PMF depth, Δ*W*_PMF_) were synonymously calculated with binding free energies from the literature to determine the influence of F691L mutation on the free energies and unbinding pathways of the protein–ligand complexes (Sun et al., 2013).

**Figure 11:**
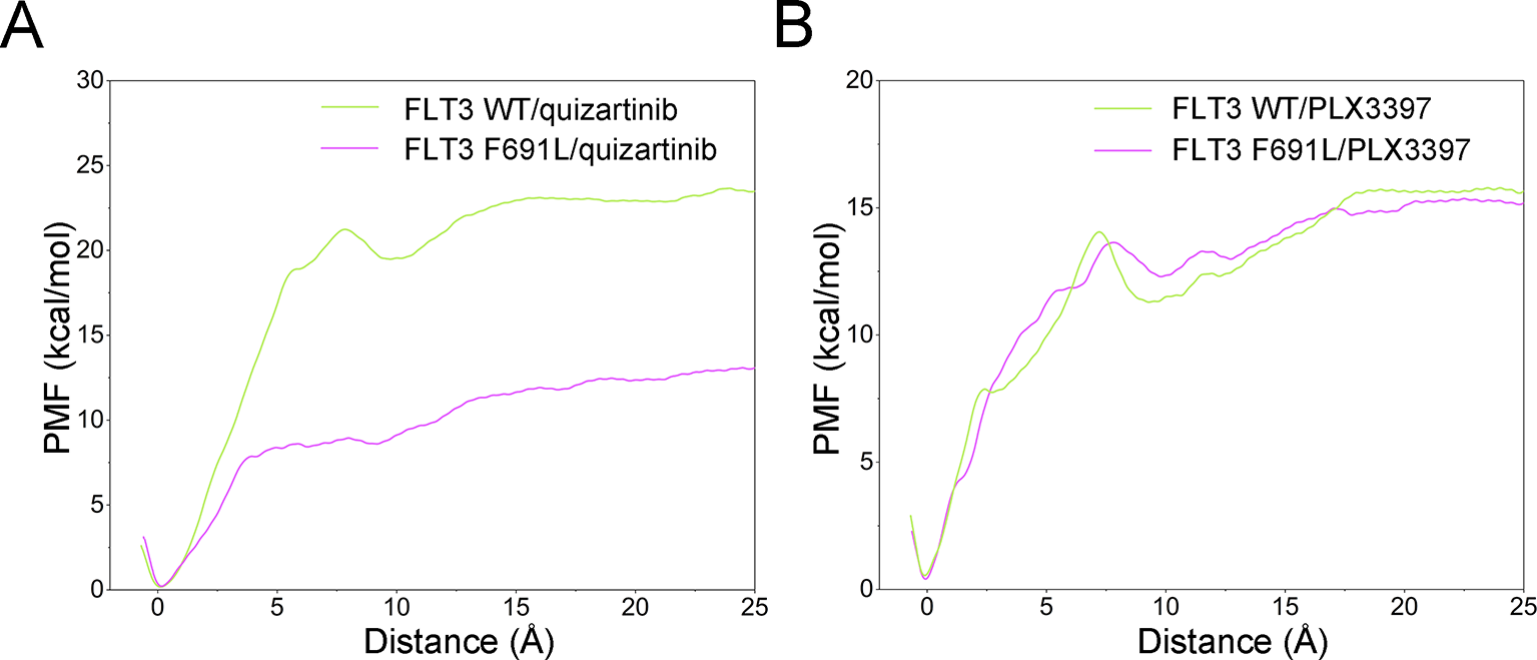
Comparison of the converged PMFs. **(A)** FLT3-WT + quizartinib and FLT3-F691L + quizartinib; **(B)** FLT3-WT + PLX3397 and FLT3-F691L + PLX3397.

The Δ*W*_PMF_ of FLT3-WT + quizartinib, FLT3-F691L + quizartinib, FLT3-WT + PLX3397, and FLT3-F691L + PLX3397 predicted by US simulations were −23.43 ± 0.47, −12.99 ± 0.55, −15.80 ± 0.47, and−15.36 ± 0.39 kcal/mol, respectively (**Table 1**). In addition, the differences of Δ*W*_PMF_ (Δ*W*_PMF_ _(WT-F691L)_) between the FLT3-WT and the FLT3-F691L for quizartinib and PLX3397 is −10.44 ± 0.51 and −0.45 ± 0.43. The Δ*W*_PMF_ and the Δ*W*_PMF_ _(WT-F691L)_ were consistent with previously reported data and correctly ranked. Thereafter, the PMF curves were utilized to provide more information on the different dissociation processes of quizartinib and PLX3397 from FLT3-WT and FLT3-F691L binding pockets (**Figure 11**). Initially, quizartinib and PLX3397 were located at the binding pockets of FLT3 WT and FLT3 F691L (RC was 0 Å). When these molecules dissociated from the binding pocket, the PMF values increased quickly (RC was 0–7 Å). Afterwards, the PMF values of quizartinib and PLX3397 increased gradually toward equivalence along with the RC (RC was 7–25 Å). During the processes of the quizartinib dissociation from FLT3-WT and FLT3-F691L, there was a much larger peak and valley in the PMF curves for FLT3-WT + quizartinib than for FLT3-F691L + quizartinib (RC was 5–12 Å, **Figure 11A**). These findings indicated the dissociation of quizartinib from FLT3-WT required higher free energy than the dissociation from FLT3-F691L. Compared with quizartinib, similar peaks and valleys were observed (RC was 5–12 Å), indicating dissociation of PLX3397 from both FLT3-WT and FLT3-F691L required considerable amount of free energy (**Figure 11B**). These observations were congruent with the previous analyses from the MM/GBSA free energy calculations and structural analysis, where the hydrogen-bond networks for quizartinib bound to FLT3-F691L were not as stably bound as FLT3-WT. In summary, the US simulations provide the details of the dissociation processes and are useful for further development of novel inhibitors to overcome the drug resistance conferred by the FLT3 F691L mutation.

## Conclusions

Previous studies have demonstrated that the FLT3-F691L mutation induces conferred resistance to quizartinib, but not to PLX3397. In this study, a set of comprehensive computational approaches were performed to explore the resistance mechanisms, both at the structural level and regarding the energy kinetics. The classical MD simulation results unambiguously demonstrated that preferential quizartinib binding to FLT3-WT over FLT3-F691L was regulated by the conformational changes of αC-helix and A-loop, which resulted in decreased ∆*E*_elec_ (decreased the hydrogen-bond interactions to key residues Glu-661 and Asp-829) and SASA. In addition, aMD simulations further supported the observations from classical MD simulations. PCA and FEL from aMD simulations suggested that FLT3 bound with quizartinib underwent large conformational changes in the presence of resistant mutation. In addition, US simulations were used to prove the predicted differences in the dissociation processes of quizartinib and PLX3397 from FLT3-WT and FLT3-F691L. The PMF depths (Δ*W*_PMF_) calculated from the US simulations were in agreement with the experimental data. Compared with FLT3-F691L, a larger energy barrier was observed from the PMF curve for quizartinib bound to FLT3-WT, implying that quizartinib dissociated more easily from FLT3-F691L than from FLT3-WT. In contrast, the dissociation processes from FLT3-WT and FLT3-F691L were similar for PLX3397. Overall, the findings in this study may prove conducive to the future design of novel potent inhibitors, which can be manipulated effectively to counter the effects of F691L gatekeeper mutation.

## Author Contributions

YS and ZX conceived and designed the experiments. YS, ZX, QZ, and BZ performed the experiments and analyzed the data. MZ and YY wrote the paper.

## Funding

This study was supported by grants from Zhejiang Provincial Natural Science Foundation of China (Grant No. LYY18H280005 and LYY19H280005).

## Conflict of Interest Statement

The authors declare that the research was conducted in the absence of any commercial or financial relationships that could be construed as a potential conflict of interest.
