# Supplementary material for: Insights Into the Resistance Mechanisms of Inhibitors to FLT3 F691L Mutation via an Integrated Computational Approach
Source: Front Pharmacol. 2019 Sep 20;10:1050. doi: 10.3389/fphar.2019.01050 (PMC6763581; doi:10.3389/fphar.2019.01050)
Supplement: Supplementary file 1 [file DataSheet_1.docx]

**Supporting information**

**Insights into the resistance mechanisms of inhibitors to FLT3 F691L mutation via an integrated computational approach**

Yunfeng Sun^1^, Zhongni Xia^1^, Qinqin Zhao^1^, Bei Zheng^1^, Meiling Zhang^1,^ *, Yin Ying^1,^ *

^1^ Department of Pharmacy, Tongde Hospital of Zhejiang Province, Hangzhou 310012, Zhejiang, China

**Corresponding author:**

Yin Ying

Department of Pharmacy, Tongde Hospital of Zhejiang Province, 234 Gucui Road, Hangzhou 310012, Zhejiang, China.

E-mail: yingyin1983@outlook.com; Phone: 86-571-89972240

Meiling Zhang

Department of Pharmacy, Tongde Hospital of Zhejiang Province, 234 Gucui Road, Hangzhou 310012, Zhejiang, China.

E-mail: zml9998@sina.com; Phone: 86-571-89972240


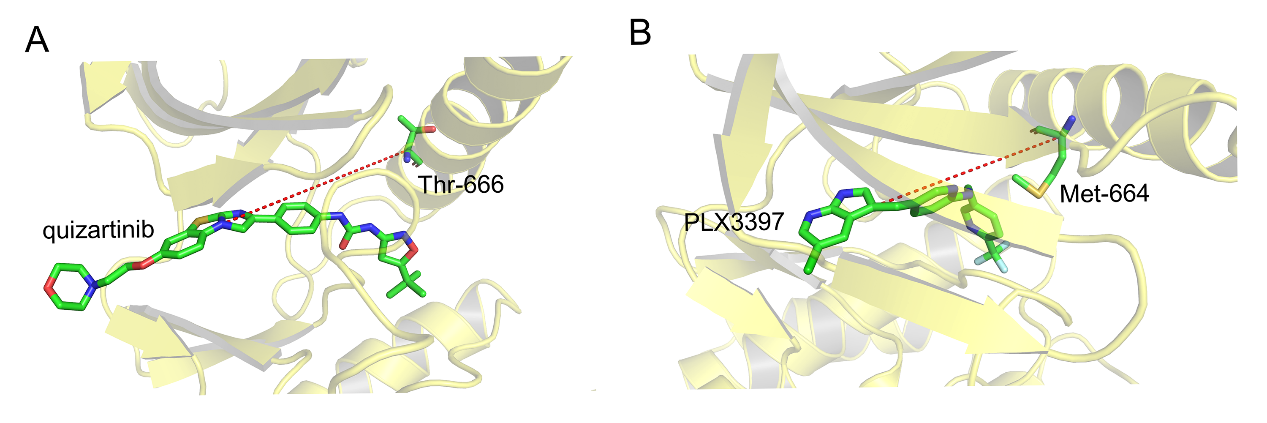


**Figure S1.** **(A)** Reaction coordinates (RCs) of umbrella sampling (US) simulations for quizartinib bound with FLT3-WT and FLT3-F691L; **(B)** RCs of US simulations for PLX3397 bound with FLT3-WT and FLT3-F691L.


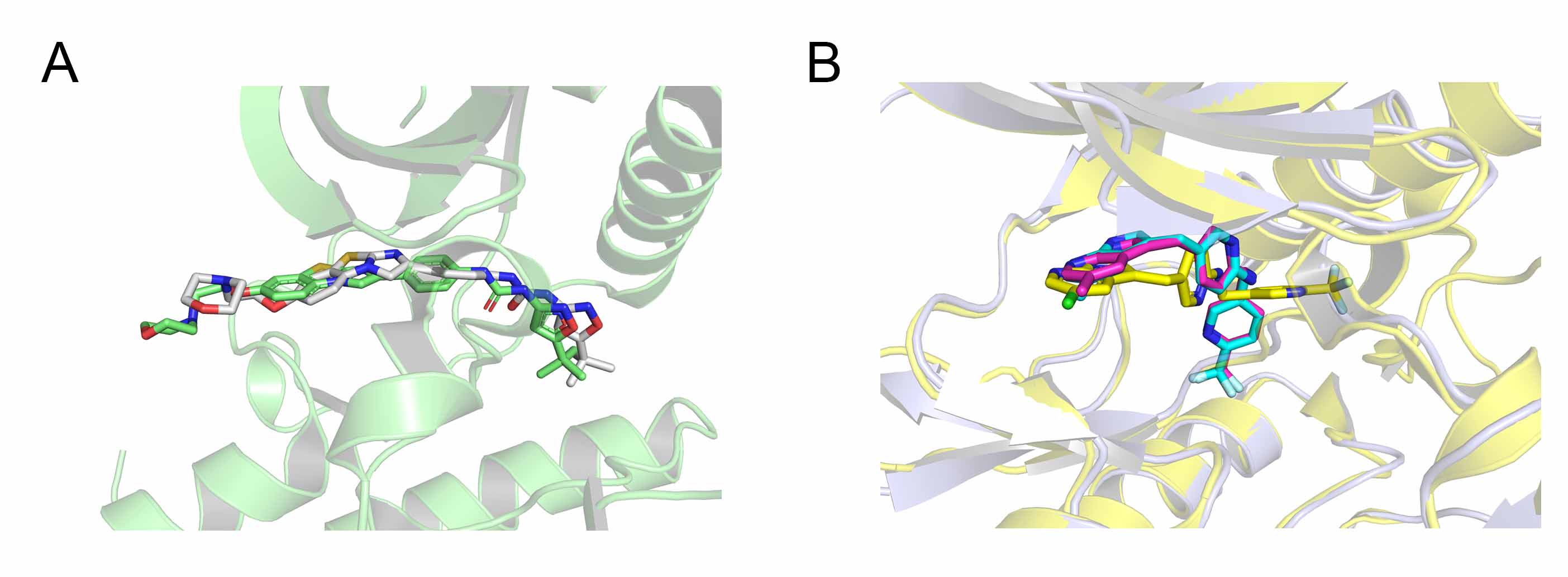


**Figure S2.** **(A)** Alignment of the crystal structure of FLT3-WT + quizartinib (green) and the predicted binding pose of FLT3-F691L + quizartinib (gray); **(B)** Alignment of the crystal structure of CSF-1R-WT + PLX3397 (yellow) and the predicted binding poses of FLT3-WT + PLX3397 (cyan) or FLT3-F691L + PLX3397 (magenta).


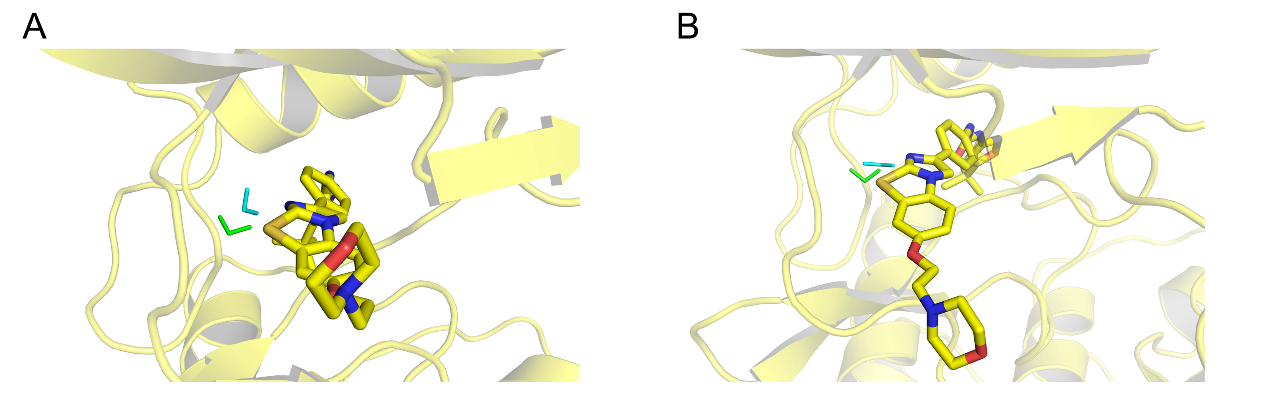


**Figure S3.** **(A)** Alignment of the water molecule in the crystal structure of FLT3-WT + quizartinib (cyan) and after classical molecular dynamics (MD) simulation (green); **(B)** Alignment of the water molecule in the crystal structure of FLT3-F691L + quizartinib (cyan) and after classical MD simulation (green);
